# P02-009 - Candle syndrome: expanding spectrum

**DOI:** 10.1186/1546-0096-11-S1-A116

**Published:** 2013-11-08

**Authors:** I Kone-Paut, A Kinoshita, H Ida, D Roybet, C Leaute-Labreze, P Pillet

**Affiliations:** 1 Paediatric Rheumatology, CeReMAI, Bicetre Hospital, University of Paris Sud, Le Kremlin Bicetre, France; 2 Human Genetics, Nagasaki University Hospital, Nagasaki, Japan; 3 Respirology, Neurology, Rheumatology, Kurume University School of Medicine, Kurume, Japan; 4 Pediatrics, CH de Hyères, Hyeres, France; 5 Dermatology, CHU de Bordeaux, Bordeaux, France; 6 Pediatric Rheumatology, CHU de Bordeaux, Bordeaux, France

## Introduction

CANDLE syndrome is an exceptional inflammatory condition starting within the first months of life, and comprising elevated fever, panniculitis with lipoatrophy, purplish and swollen eyelids, arthralgia, and developmental retardation. Most patients carry homozygous mutations in the *PSMB8* gene that impair the assembly of the immunoproteasome (iP) and lead to interferon g deregulation. Since now, 39 published case reports under various acronyms have shown clinical and genetic heterogeneity, suggestive of various mechanisms underlying this very severe condition. We present two new cases enlarging the spectrum of CANDLE phenotype.

## Case report

**1**: A Sicilian girl, born at term in 1986 with microcephaly, skin panniculitic rash since 2 weeks, hepatosplenomegaly, microcytic anemia, leucopenia with extreme lymphopenia, myelemia, thrombopenia, elevated ESR, liver enzymes and ANA, first suspected with neonatal lupus. She developped recurrent otitis media and pneumonitis. Following years, recurrent fever, severe growth and mental retardation (cerebral calcification, seizures), purplish swollen eyelids, rashes mimicking dermatomyositis, joint contracture, severe lipoatrophy, diabetes requiring insulin therapy, dyslipidemia and finally severe hypertension and giant aortic aneurysm that killed her at the age of 16y. Proteasome exons PMSB1-10, PSME1-3, PSMA1-6 were screened and did not reveal any mutation. No other biologic material is available for IFNy investigation. **2:** A French girl, born in 2009, onset at 2 months, hepatosplenomegaly, seizures, panniculitis, fever with peaks, swollen eyelids and sometimes hands and feet, joint contracture and lipoatrophy increasing with time. She also has marked leucopenia, microcytic anemia and elevated liver enzymes. Extensive work up to rule out other condition not contributive. *PMSB8* gene analyses are ongoing.

## Discussion

Case 1 could be considered as an extreme severity phenotype of CANDLE, as the developmental retardation was intriguing (final height 87cm), immunodeficiency and autoimmunity were both present with also important metabolic disturbances, finally she died early at 18y. Case 2 shares many features of CANDLE, even the skin biopsy was not typical (immature white cells not retrieved). She may have mild developmental retardation. These 2 patients confirm that other causes outside the proteasome may cause CANDLE phenotype.

## Disclosure of interest

None declared.
